# Exopolysaccharide is required by *Paraburkholderia phytofirmans* PsJN to confer drought-stress tolerance in pea

**DOI:** 10.3389/fmicb.2024.1442001

**Published:** 2024-08-09

**Authors:** Cahya Prihatna, Qing Yan

**Affiliations:** Plant Sciences and Plant Pathology Department, Montana State University, Bozeman, MT, United States

**Keywords:** *Paraburkholderia phytofirmans* PsJN, *Pisum sativum*, drought tolerance, exopolysaccharide, nodulation

## Abstract

*Paraburkholderia phytofirmans* PsJN is a plant symbiotic bacterium that can colonize a broad spectrum of plant hosts and frequently shows beneficial effects on plant growth. Exopolysaccharide (EPS) is known to be important in plant-bacteria interactions. Previously, we reported that EPS is required for PsJN to survive from drought stress and colonize in pea (*Pisum sativum*) under drought condition. However, whether EPS is necessary for PsJN to promote plant growth remains unknown. In this work, a comparative study was conducted between the wild-type PsJN and its ∆*bceQ* mutant that lacks EPS to investigate the role of EPS in PsJN to confer drought-stress tolerance on pea plant. Our results showed that wild type PsJN, but not the ∆*bceQ* mutant, promoted pea seed germination and seedlings growth under drought stress. Pea plants inoculated with the wild type PsJN had a higher level of drought tolerance, as shown by a better vegetative growth and enhanced nodule formation, than plants inoculated with the ∆*bceQ* mutant. Moreover, EPS plays a role in the plant colonization under drought stress, because the ∆*bceQ* mutant was unable to colonize pea seeds and roots as effectively as the wild type PsJN. Further, expression of the EPS biosynthesis genes in the *bceOVN* operon of the wild type PsJN was induced by the presence of glucose. Overall, this study demonstrated that PsJN can promote pea plant growth under drought conditions and EPS is required for PsJN to confer beneficial effects to host plant.

## 1 Introduction

Plants are constantly exposed to a wide range of environmental stresses that limit their productivity. Among these, drought is a major environmental stress that represents a major challenge in crop growth and productivity worldwide. It has been known that plants interact with a wide range of beneficial bacteria that can alleviate the deleterious effects caused by the environmental stresses (Esmaeel et al., 2018). Several beneficial bacteria are shown to induce drought stress tolerance in some plants such as *Pseudomonas aeruginosa* GGRJ21 in mungbean, phenazine-producing rhizobacteria in wheat, *Pseudomonas* spp. in finger millet, and *P. putida* and *Bacillus amyloliquefaciens* in chickpea (Sarma and Saikia, 2014; Kumar et al., 2016; Chandra et al., 2018; Mahmoudi et al., 2019). It is suggested that under stress conditions, plants become more dependent on beneficial microorganisms to extend their capability of resisting stress (Kavamura et al., 2013).

*Paraburkholderia phytofirmans* strain PsJN is a plant symbiotic bacterium and has been shown to promote growth and fitness of several plant species. PsJN was shown to improve drought tolerance in maize and wheat (Naveed et al., 2014a,b) and promote plant growth under other abiotic stresses such as salinity in quinoa (Yang et al., 2020), low temperature (4°C) on grapevine (Ait Barka et al., 2006), high temperature (32°C) on tomato (Issa et al., 2018), and freezing temperatures on *Arabidopsis thaliana* (Su et al., 2015). In addition, PsJN also protects plants from biotic stresses, such as suppression of virulent strain of *P. syringae* in *Arabidopsis thaliana* (Timmermann et al., 2017), Pierce’s disease of grape in the field (Lindow et al., 2024), and crown gall disease caused by *Allorhizobium vitis* in grapevine (Trong et al., 2022). Possible mechanisms by which PsJN exert beneficial effects include direct modulation of plant hormones (Pieterse et al., 2012), nutrient and resource acquisitions (Naveed et al., 2014a), or through production of secondary metabolites (Esmaeel et al., 2018) and induction of host systemic resistance (Pieterse et al., 2014), all leading to improved adaptability to diverse stresses.

The ability of PsJN to protect many plants from different abiotic and biotic stresses makes it a model bacterium to study the molecular mechanisms of symbiotic plant-bacteria interactions (Esmaeel et al., 2018). For example, functional analysis of *acdS* gene, encoding 1-aminocyclopropane-1-carboxylate deaminase that lowers plant ethylene levels, showed that it plays a role in plant growth promotion by PsJN (Sun et al., 2009). Additionally, bacterial degradation of indole-3-acetic acid by PsJN was found to play a major role in plant-growth-promoting traits and is necessary for efficient rhizosphere colonization (Zúñiga et al., 2013). Further research revealed that the auxin degradation pathway of PsJN is governed by an *iac* gene cluster, which is regulated by a putative two-component regulatory system and a LysR-type regulator (Donoso et al., 2016).

Bacterial EPS is important for bacterial attachment and modification of surface properties that determine the establishment of effective symbiosis and stress adaptation (Janczarek et al., 2015). EPSs are carbohydrate polymers of highly variable composition and structure found outside cells (Schmid et al., 2015). EPS, along with flagella, pili, bacterial exudates, and signaling molecules, have been shown to play a role in the formation of biofilms and the early steps of bacterial colonization on plants (Rodríguez-Navarro et al., 2007). The genome of PsJN harbors a *bce* gene cluster consisting of 19 genes (*bceABCDEFGHIJKNVOPQRST*) that encodes components for cepacian EPS biosynthesis (Weilharter et al., 2011). We previously reported that EPS is required for PsJN to survive under different stresses such as desiccation, UV damage, salt and iron stresses, and bacteriophage infection and plays a role in the colonization on camelina (*Camelina sativa*) and pea (*Pisum sativum*) (Fu and Yan, 2023). However, the role of EPS in the beneficial effects of PsJN on plants under stressful condition has not been investigated. In this study, we tested whether PsJN can promote the growth of pea plants under drought stress. The role of EPS in the PsJN-mediated pea drought tolerance was investigated through a comparative study between the wild type PsJN and its mutant that lacks EPS production. The role of EPS for PsJN to survive under osmotic stress and the expression profiles of the EPS biosynthesis genes were also investigated.

## 2 Materials and methods

### 2.1 Bacterial strains, plasmids, culture conditions, and plant materials

Bacterial strains and plasmid constructs used in this study are listed in Table 1. *Paraburkholderia phytofirmans* PsJN and its derivatives were cultured in King’s B (KB) medium (King et al., 1954), or potato dextrose agar/broth (PDA/PDB, Difco™ United States) at 28°C. *Escherichia coli* strains were cultured on Luria broth/agar (LB/LA, Difco™ United States) at 37°C. Common peas (*P. sativum*) var. Carousel was used as the plant materials.

**Table 1:** Bacterial strains, plasmids, and oligonucleotides used in this study.

Strains, plasmids	Remarks^*^	Source
*Paraburkholderia phytofirmans*		
LK583	Spontaneous rifampicin-resistant mutant of the wild type PsJN (LK545), Rif^r^	This study
LK754	Δ*bceǪ* mutant of PsJN, made by introducing the deletion construct pEX18TC-Δ*bceǪ* into wild type PsJN (LK583), Rif^r^	Fu and Yan (2023)
LK838	LK583 carrying pPROBE-NT that harbors 801 bp *bceOVN* promoter fused to *gfp* (p*bceOVN*_promoter_:*gfp*), Kan^r^	This study
LK839	LK583 carrying pPROBE NT that harbors 881 bp *bcePǪR* promoter fused to *gfp* (p*bcePQR*_promoter_:*gfp*), Kan^r^	This study
LK771	Wild type PsJN (LK583) containing the empty vector pPROBE-NT, Kan^r^	This study
*Escherichia coli*		
LK768	NEB^®^ 5-alpha carrying pPROBE-NT that harbors 801 bp *bceOVN* promoter fused to *gfp* (p*bceOVN*_promoter_:*gfp*), Kan^r^	This study
LK769	NEB^®^ 5-alpha carrying pPROBE-NT that harbors 881 bp *bcePǪR* promoter fused to *gfp* (p*bcePQR*_promoter_:*gfp*), Kan^r^	This study
Plasmids		
pPROBE-NT	Plasmid containing promotorless *gfp* reporter gene. pBBR1 backbone, Kan^r^	Miller et al. (2000)
p*bceOVN*_promoter_:*gfp*	pPROBE-NT that harbors 801 bp *bceOVN* promoter fused to *gfp*, Kan^r^	This study
p*bcePQR*_promoter_:*gfp*	pPROBE-NT that harbors 881 bp *bcePǪR* promoter fused to *gfp*, Kan^r^	This study
Oligonucleotides	DNA sequences (5′–3′)	
*bceO* promoter F	tataAGCTTTTGAACGTGAAACGG	
*bceO* promoter R	atagaaTTCAACGCGTGCTGCGCGAAC	
*bcePQ* promoter F	tataagcTTCAACGCGTGCTGCGCGAAC	
*bcePQ* promoter R	atagaaTTCAACGCGGTTTCCGCATG	

^*^Rif^r^: rifampicin resistance at 100 μg/mL; Kan^r^: kanamycin resistance at 50 μg/mL.

### 2.2 Bacterial inoculum and plant inoculation for drought-stress tolerance assay

A spontaneous rifampicin-resistant derivative of *P. phytofirmans* PsJN namely LK583 was used in all studies and is hereafter referred to as wild-type PsJN. The ∆*bceQ* mutant is a derivate of PsJN and was generated in our previous work (Fu and Yan, 2023). Inoculum of each bacterial strain was prepared by growing them separately on KB agar for 48 h at 28°C. Cells were collected from the agar plates and washed three times into 1 mL using sterilized deionized water. Optical density of the cells was adjusted to OD_600_ of 1.0 in 10 mL of sterile water. Seeds of peas were surface sterilized using 10% (v/v) of sodium hypochlorite for 2 min twice. The seeds were then immersed in the bacterial cell suspension for 3 h without agitation. As a control, some seeds were immersed only in sterile water without bacterial cells.

### 2.3 Seed germination assay under osmotic stress conditions

Wild type PsJN and ∆*bceQ* mutant were grown on KB plates for 2 days at 28°C and the cells were scraped from the plates, resuspended in sterile deionized water, and optical density of the cells was adjusted to OD_600_ of 1.0 in 10 mL of sterile deionized water. Cell suspensions at OD_600_ of 1.0 were also prepared in 10 mL of polyethylene glycol (PEG) 6000 (Sigma-Aldrich, United States) solutions at different concentrations, i.e., 2% PEG, 4% PEG, and 6% PEG.

Pea seeds were surface-sterilized using sodium hypochlorite 10% (v/v) for 2 min twice and immersed in the bacterial suspension in water and in solutions containing PEG at different concentrations for 3 h without agitation. After 3 h, the seeds were transferred onto Petri dishes that had four sheets of sterile brown tissue paper moistened with 6 mL of sterile water and different concentrations of PEG, as specified earlier.

The seeds were put in the dark at room temperature and the number of germinated seeds and root length were observed daily for 7 days. Ten seeds were used for each Petri dish and three Petri dishes were used in each treatment. The experiment was repeated two times independently.

### 2.4 Drought-stress tolerance assay

Soil used for these experiments was a pasteurized 50:50 mixture of Montana State University (MSU) Mix and Sunshine Mix No. 1 (pH 7.3; NO_3_-N 68 ppm; NH_4_-N 3 ppm; PO_4_-P 3 ppm; potassium 47 ppm; magnesium 118 ppm; calcium 570 ppm; sodium 31 ppm). The soil was put into 4-cm-wide, 20-cm-tall, and slender pots. The pots were kept in a greenhouse that was maintained at 22/18°C (day/night), a humidity level of 42%, and a photoperiod of 16/8 h light/darkness, at the Plant Growth Center, Bozeman, Montana.

Each of the inoculated seed was sown into one pot. Therefore, for each treatment, there were 21 pot replicates (1 seed per pot). Regular watering was provided based on the moisture level of the soil. Drought treatment commenced when the seedlings had two successive nodes, which took 14 days since seed sowing. At this stage, watering was ceased for 7 days, and resumed with the following watering regime. The field capacity (non-drought stress) for each pot was reached when the soil was watered with 35 mL of water per pot. For drought treatment, water was given at 60% field capacity (21 mL of water per pot). All bacteria-inoculated seedlings were watered at 60% field capacity, including a negative control that consists of uninoculated seedlings watered at 60% field capacity. A positive control was also included in this experiment consisting of uninoculated seedlings watered at 100% field capacity. Following the 7-day watering stoppage, watering was performed once every 3 days using the water levels mentioned above.

The plant height was measured every 7 days starting at 7 days after drought treatment commenced (at the end of 7-day without watering) until 21 days after drought treatment was started. At the end of the experiment (21 days post drought commencement), the seedlings were uprooted and the number of nodules in each root system was counted. The experiment was repeated two times independently.

### 2.5 Enumeration of bacterial population on the rhizoplane

The surface-sterilized pea seeds were inoculated with bacteria as described in section 2.2. To enumerate bacterial cells adhering to the seed surfaces, the inoculated seeds were air-dried for 1 h in a laminar flow hood and then one seed was immersed in 10 mL of sterile water in a 50-mL canonical tube. The immersed seeds were shaken vigorously for 30 min. The bacterial suspension was then collected and its serial dilutions were spread on KB agar plates supplemented with rifampicin 100 μg/mL. The agar plates were incubated for 3 days at 28°C and the number of colonies was counted. Three seeds were used in each treatment of the experiment. The experiment was repeated two times independently.

The root-associated soil samples were collected by the method described by Edwards et al. (2015) with some modifications. We specifically targeted the bacterial populations that tightly adhered at the rhizoplane. Briefly, plants were uprooted at the end of drought experiment. The excess bulk soils were manually shaken off from the roots. The roots were then cut into three sections, i.e., the closest to the crown as a proximal fragment; middle root fragment; and the farthest from the crown as a distal fragment. On average, the length of root in each section was four centimeters. The root fragment was put into a 50-mL conical tube containing 40 mL of sterile water. The tube was shaken to remove loosely attached soils from the root. This washing step was repeated three times, and the final wash of water was added at 18 mL and the tubes were shaken vigorously for 30 min. The water was then collected and serial dilution was spread on KB agar plates supplemented with rifampicin 100 μg/mL. Cycloheximide (100 μg/mL) was also added to the medium to exclude possible fungal contaminations. The agar plates were incubated for 3 days at 28°C and the number of colonies was counted. The experiment was repeated two times independently.

### 2.6 *In vitro* osmotic-stress tolerance assay in PsJN

PsJN and ∆*bceQ* mutant were grown on PDA for 2 days at 28°C. Cells were harvested and resuspended in 1 mL of sterile deionized water. The cells were washed three times and cell density was adjusted to OD_600_ of 1.0 in 1 mL of sterile deionized water.

The cell suspension (20 μL) of each strain was spread on PDA supplemented with different concentrations of PEG, i.e., PEG 0%, PEG 0.5%, PEG 1.0%, and PEG 1.5%. The plates were incubated at 28°C for 3 days before the results were recorded. The experiment was repeated two times independently.

### 2.7 Construction of GFP reporter strains and EPS gene expression study

To measure the promoter activity of *bceOVN* operon, a 801-bp DNA of intergenic region between *bceOVN* and *bcePQR* operons containing *bceOVN* promoter was amplified from the wild-type PsJN genome using oligonucleotide pair *bceO* promoter F/*bceO* promoter R (Table 1), digested with *Eco*RI and *Hin*dIII, and ligated into pPROBE-NT to generate reporter construct p*bceOVN*_promoter_:*gfp* that contains *bceOVN*_promoter_:*gfp* transcriptional fusion. Similarly, a 881-bp DNA of intergenic region between *bcePQR* and *bceOVN* operons containing *bcePQR* promoter was amplified using oligonucleotide pair *bcePQ* promoter F/*bcePQ* promoter R (Table 1) to generate a fragment that has an opposite transcriptional orientation to the above 801-bp DNA of intergenic region, digested with *Eco*RI and *Hin*dIII, and ligated into pPROBE-NT to generate reporter construct p*bcePQR*_promoter_:*gfp* that contains *bcePQR*_promoter_:*gfp* transcriptional fusion.

The generated reporter constructs p*bceOVN*_promoter_:*gfp* and p*bcePQR*_promoter_:*gfp* were transformed into wild-type PsJN by electroporation. The transformed PsJN harboring each reporter construct was grown on PDA plates supplemented with kanamycin 50 μg/mL. To test the GFP activity of the reporter strains, PsJN containing the reporter constructs was cultured on M9 agar media supplemented with varying concentrations (0.4, 0.8, 1.6, and 3.2%) of glucose. The cultures were grown at 28°C. PsJN carrying an empty vector pPROBE-NT was used as a control.

The GFP intensity of the cultures was measured at 24, 48, and 72 h of growth on the agar. Specifically, bacterial cells were collected from the culture plates and washed into 1 mL in sterile deionized water. GFP activity and the cell density (OD_600_) of the bacterial cell suspensions was measured using a SPARK^®^ multimode Microplate Reader (TECAN, Switzerland) with an excitation wavelength at 485 nm and emission wavelength at 535 nm. The relative GFP intensity was then calculated by normalizing the GFP intensity against the OD_600_ and then subtracting the normalized GFP intensity with GFP reading from the bacterial strains with the empty vector. Each treatment of the experiment has at least four replicates. The experiment was repeated two times independently.

### 2.8 Statistical analysis

All statistical analysis was performed using R version 4.3.2. One-way ANOVA was used to analyze the difference between the means of groups (*p <* 0.05) with further adjustment using Tukey’s HSD *post hoc* test.

## 3 Results

### 3.1 PsJN enhanced pea seed germination under osmotic stress condition

**Figure 1:**
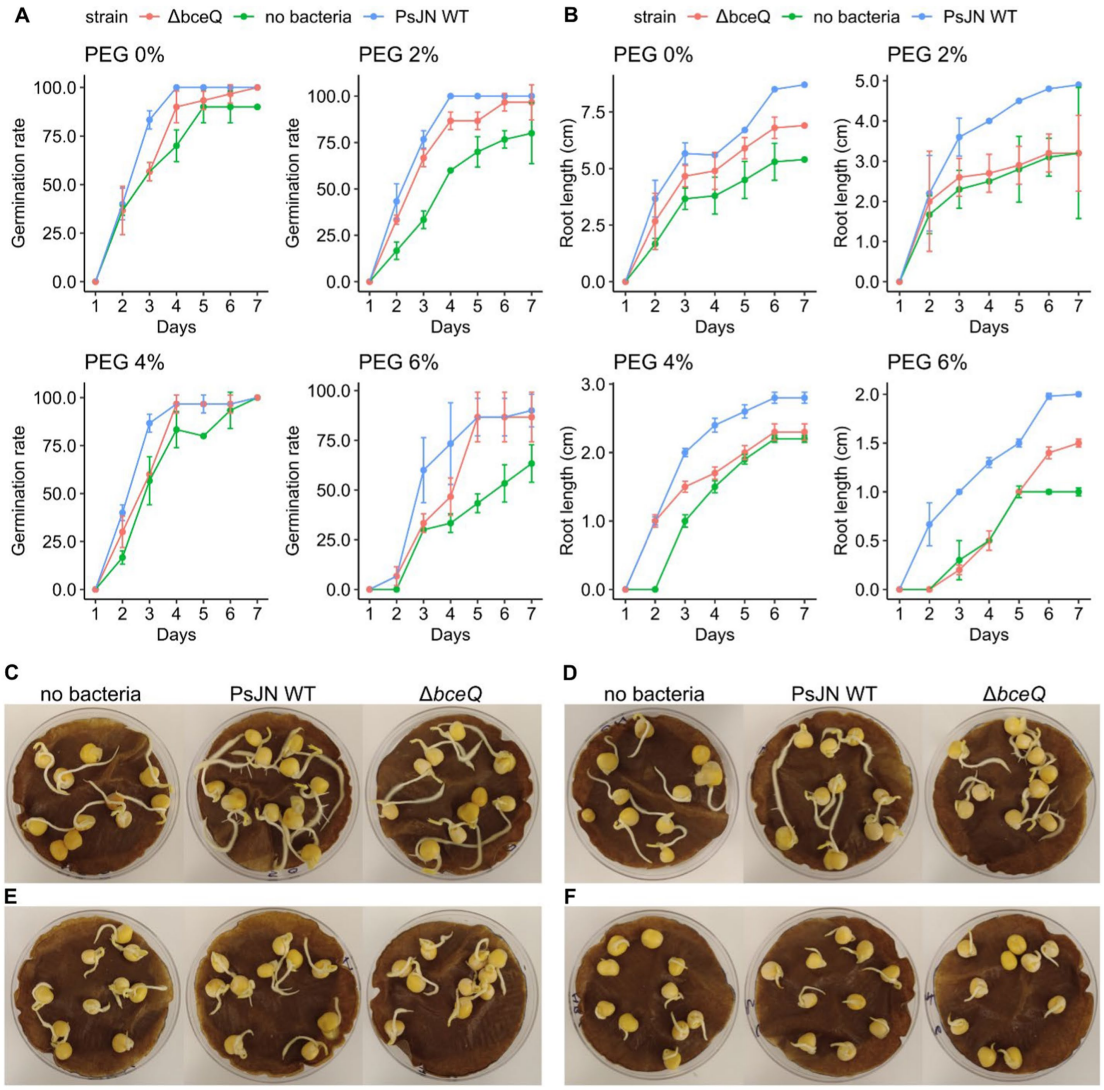
Germination rate and seedling root length in peas inoculated with *Paraburkholderia phytofirmans* PsJN under osmotic stress induced by polyethylene glycol (PEG). **(A)** Germination rate of pea seeds inoculated with wild type PsJN and ∆*bceQ* mutant under normal condition (no PEG), and osmotic stress induced by PEG 2, 4, 6%. **(B)** Root length of the germinated pea seeds. **(C–F)** A representative photograph of germinated pea seeds under normal osmotic condition (no PEG) **(C)**, and under osmotic stress at PEG 2% **(D)**, PEG 4% **(E)**, and PEG 6% **(F)**. Photograph was taken at 7 days after inoculation. Observation on germination and root length was conducted daily for 7 days. Data were obtained from three replicates of 10 seeds for each treatment in one experiment, and the experiment was repeated two times.

Pea seed germination assays showed that wild type PsJN promoted seed germination at normal osmotic conditions (without PEG) and at hyperosmotic conditions (PEG 2, 4, 6%) (Figure 1). Most seeds germinated 2 days after inoculation. Relative to no bacteria treatment, wild type PsJN-treated seeds had a higher level of germination rate at all PEG concentrations. Overall, this trend continued over the 7-day course of the experiment under both normal and hyperosmotic conditions. Interestingly, the beneficial effect of wild type PsJN in promoting seed germination was more pronounced at the higher level of osmotic stress (PEG 6%), as shown by the result that wild type PsJN-treated seeds had a germination rate (90%), which is higher compared with seeds without bacterial treatment (60%) (Figures 1A,F).

In addition, wild type PsJN also promoted the seedling growth under normal and hyperosmotic stress (Figure 1B). Root lengths of the seedlings inoculated with wild type PsJN were longer than the uninoculated seedlings (*p* = 0.015). For example, at 6% PEG, the average root length of wild type PsJN-treated seedlings was 2 cm, whereas root length of uninoculated seedlings was 1 cm. This root elongation induced by wild type PsJN was also observed in the control condition in which PEG was not added. Moreover, the roots of wild type PsJN-inoculated seedlings had more lateral roots than seedlings without bacterial inoculation, particularly at PEG 2% and no PEG (Figures 1C,D).

In contrast, seeds inoculated with ∆*bceQ* mutant had a lower germination rate and shorter roots than seeds inoculated with the wild type PsJN (Figure 1). The difference in germination rate between seeds treated with wild type PsJN and ∆*bceQ* mutant was evident at earlier time points (2–4 days post inoculation) (Figure 1A). The difference in germination rate was not significant at later stages as most of the ∆*bceQ* mutant-treated seeds germinated eventually (*p* = 0.89). The root length of ∆*bceQ* mutant-treated seedlings was shorter than the wild type PsJN-treated seedlings under the osmotic stresses (*p* = 0.03) (Figure 1B). Moreover, compared to the wild type PsJN-treated seedlings, the ∆*bceQ* mutant-treated seedlings also had less lateral roots (Figures 1C,D).

Overall, these results indicate that wild type PsJN can promote pea seed germination and root development under both normal and osmotic stress conditions. Mutation of the EPS biosynthesis gene *bceQ* reduced the beneficial effect of PsJN.

### 3.2 PsJN promoted drought tolerance in peas

**Figure 2:**
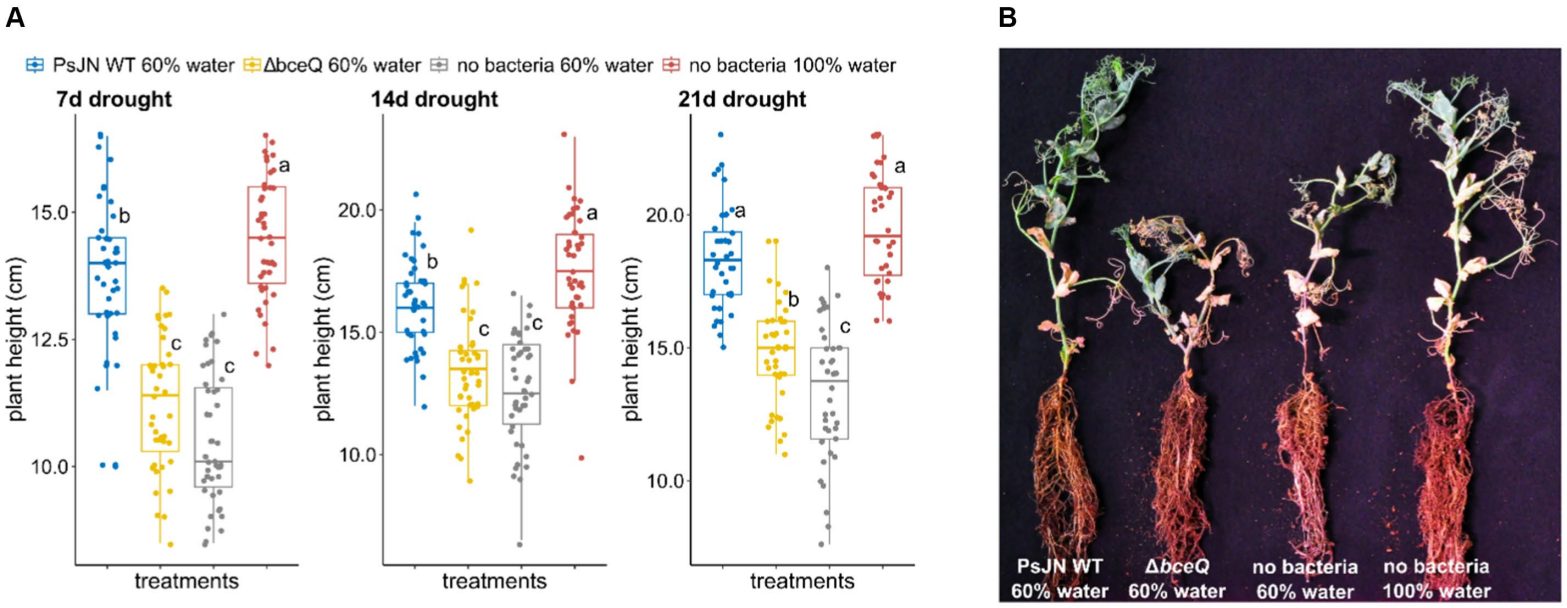
Drought tolerance assay of common peas (*Pisum sativum*) under greenhouse conditions. **(A)** Plant height was measured at 7, 14, and 21 days after drought treatment commenced. Drought treatment was conducted by watering the plants at 60% field capacity starting when the seedlings produced two nodes (14 days after seed sowing). **(B)** Photograph of representative pea plants inoculated with wild-type *Paraburkholderia phytofirmans* PsJN, ∆*bceQ* mutant, watered at 60% water capacity (drought condition), and seedlings without bacterial inoculation watered at 60 and 100% field capacities. Photograph was taken at 21 days after drought treatment commenced. Each treatment had 21 plant replicates. The experiment was repeated three times independently. Variance in variables was analyzed using one-way ANOVA and *post hoc* adjustment was conducted using Tukey’s HSD. Boxes topped by the same letter are not significantly different at the *p* < 0.05 level. Statistical analysis was performed using R.

Under drought condition, plants inoculated with wild type PsJN had higher average of plant height than plants without bacterial inoculation (*p* = 1.2 × 10^−7^) (Figure 2). The growth promotion of these plants by wild type PsJN was observed as early as 7 days after drought treatment (Figure 2A). Throughout the 3-week observation the pattern in plant height was relatively constant in which the height of plants inoculated with the wild-type PsJN under drought condition was similar to the plants watered at field capacity without bacterial inoculation (*p* = 0.07).

In contrast, no growth promotion effect was observed in pea plants inoculated with ∆*bceQ* mutant under drought conditions. Both plants inoculated with the ∆*bceQ* mutant and plants without bacterial inoculation at 60% field capacity were significantly shorter than wild type PsJN-inoculated plants and the plants watered at field capacity.

### 3.3 PsJN promoted the development of nodules in peas under drought condition

**Figure 3:**
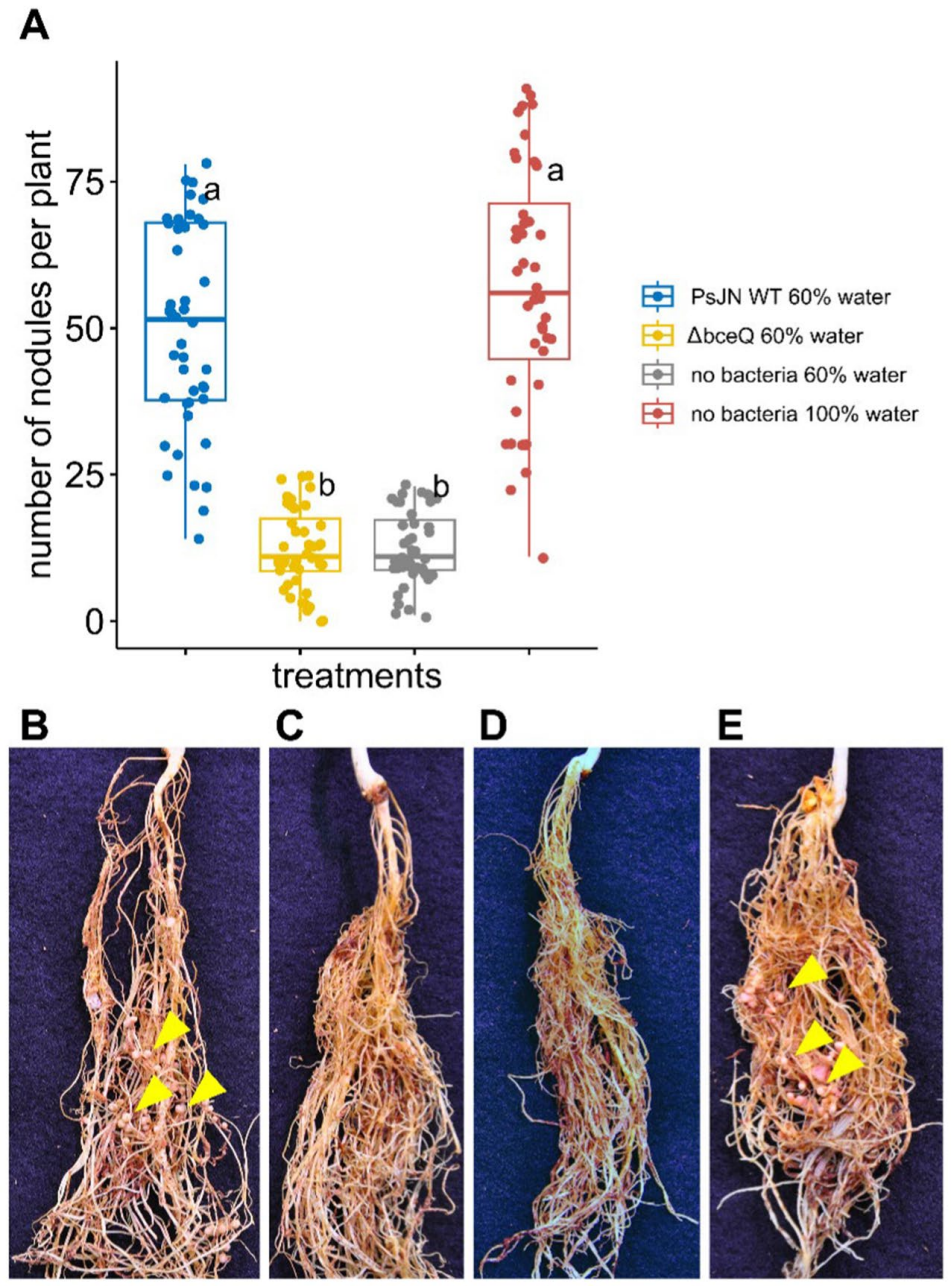
The effect of wild type *Paraburkholderia phytofirmans* PsJN and its ∆*bceQ* mutant on nodulation of common peas (*Pisum sativum*) under drought conditions. **(A)** The number of nodules formed in the roots per plant at 21 days after drought treatment commenced. **(B)** Photograph of roots from a pea plant watered at 60% field capacity (drought condition) inoculated with wild type PsJN. **(C)** Roots of a plant under drought condition inoculated with ∆*bceQ* mutant. **(D)** Roots of a pea plant under drought condition without bacterial inoculation. **(E)** Roots of a pea plant watered at 100% field capacity without bacterial inoculation. Yellow arrowheads show several examples of nodules formed in the roots. Photograph was taken at 21 days after drought treatment commenced. There were 21 plant replicates for each treatment and the experiment was repeated three times independently. Variance in variables was analyzed using one-way ANOVA and *post-hoc* adjustment was conducted using Tukey’s HSD. Boxes topped by the same letter are not significantly different at the *p <* 0.05 level. Statistical analysis was performed using R.

In addition to promoting pea seed germination and seedling growth, wild type PsJN also induced nodule development in pea roots under drought conditions. Roots of plants inoculated with wild type PsJN formed significantly more nodules than roots of uninoculated plants (*p* = 2.1 × 10^−8^) (Figure 3A). The average number of root nodules in wild type PsJN-inoculated plant was comparable to the plants watered at field capacity without bacterial inoculation (*p* = 0.09). The wild type PsJN-treated plants produced around 52 nodules per plant and the plants with normal treatment produced around 55 nodules per plant. The nodules that were formed in roots of wild type PsJN-inoculated plants were fully developed and similar to the nodules that were formed in uninoculated plants with normal watering (Figures 3B,D).

However, the number of nodules produced by ∆*bceQ* mutant-treated plant was significantly lower (~12 nodules per plant) than wild type PsJN-treated plants under drought stress (*p* = 2.3 × 10^−8^). The level of nodule formation in the ∆*bceQ* mutant-treated plants was similar to the untreated plants when they were grown under drought stress (*p* = 0.99) (Figure 3). These results suggest that drought stress decreased the root nodule development and that wild type PsJN promoted the nodule development in an EPS-dependent manner.

### 3.4 *bceQ* influences root colonization by PsJN

**Figure 4:**
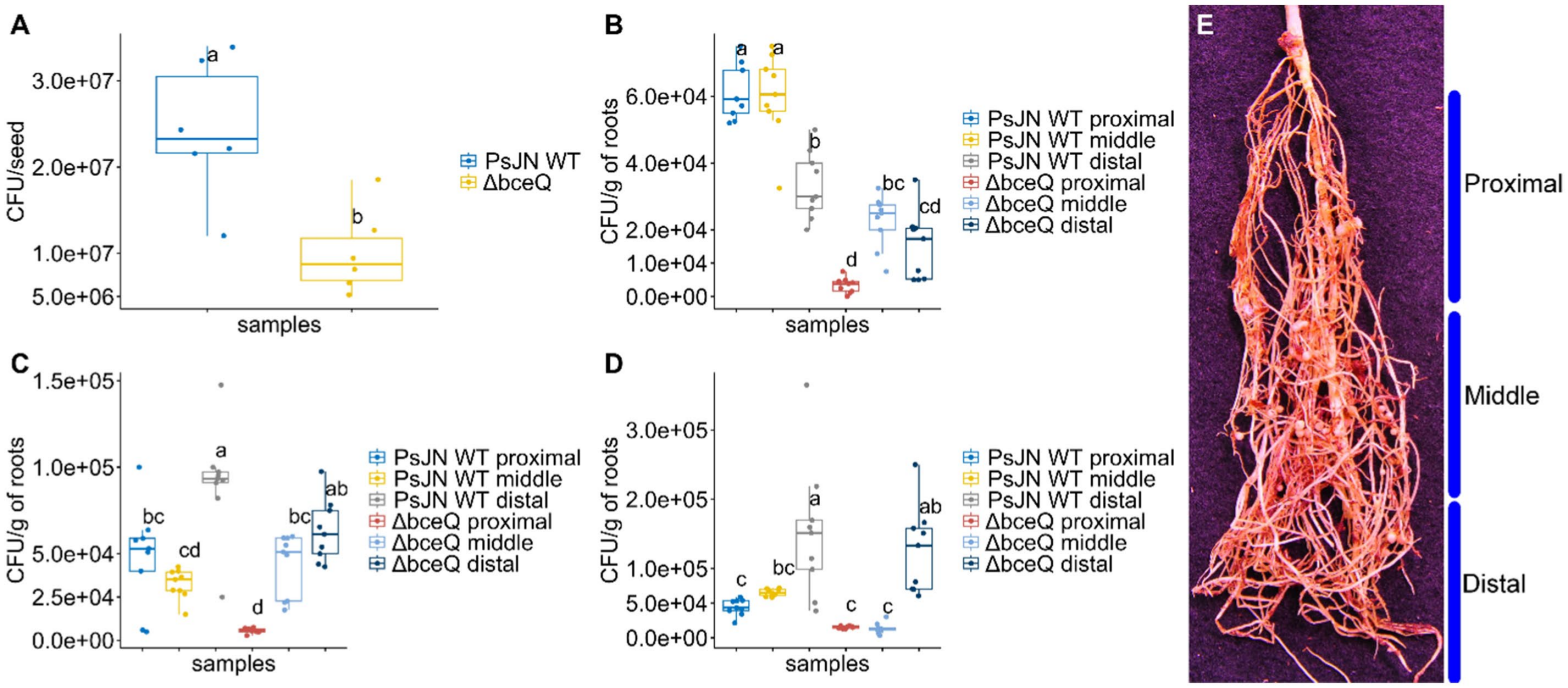
The population of *Paraburkholderia phytofirmans* PsJN and its ∆*bceQ* mutant on the seeds and in the rhizoplane of peas under drought conditions. **(A)** Population of wild type PsJN and ∆*bceQ* mutant cells recovered from the surface of the seeds following seed inoculation. **(B–D)** Population of wild type PsJN and ∆*bceQ* mutant cells recovered from the rhizoplane of peas at 7 days **(B)**, 14 days **(C)**, and 21 days **(D)** after drought treatment commenced. **(E)** Approximation of sectioning of root system into proximal, middle, and distal regions. Population of bacteria from the seeds was obtained from three seeds for each treatment and the experiment was repeated two times. To determine the bacterial population in the rhizoplane, the root samples were collected from three pea plants randomly and the root system was divided into three sections, i.e., proximal, middle, and distal regions. The experiment was repeated three times. One-way ANOVA was used to compare the variances between the means of different treatments and post-hoc adjustment was conducted using Tukey’s HSD. Boxes topped by the same letter are not significantly different at the *p* < 0.05 level. Statistical analysis was performed using R.

Exopolysaccharide is known to play a role in bacterial attachment on biotic and abiotic surfaces. We proposed that EPS is required for wild type PsJN to effectively colonize on pea seed and/or root surfaces. To test this hypothesis, the cells of wild type PsJN and the ∆*bceQ* mutant were recovered from the bacteria-treated seeds and seedlings at different time points. Our results showed that the ∆*bceQ* mutant’s population on the seed surfaces was only around half (1.0 × 10^7^ CFU/seed) of the wild type PsJN’s population (2.2 × 10^7^ CFU/seed) (*p* = 0.03) (Figure 4A).

Likewise, the population of wild-type PsJN in the rhizoplane was overall significantly higher than the ∆*bceQ* mutant (Figures 4B–D). The population of wild type PsJN was higher than ∆*bceQ* mutant in each of the tested root zones including proximal, middle, and distal. Interestingly, there were shifts in the dynamics of bacterial population within the root zones throughout the drought treatment. Populations of the wild type PsJN were found mostly at the proximal zone of the roots (closer to the crown) at 7 days post-drought treatment, and shifted to the zones further away from the crown toward the distal zone (closer to the root tips) at 14 and 21 days post-drought treatment. A similar pattern of spatial shift was also observed in the population of ∆*bceQ* mutant although the population size was overall lower than the wild type PsJN especially at the early stage of the root colonization.

### 3.5 *bceQ* plays a role in tolerance to osmotic stress in PsJN

**Figure 5:**
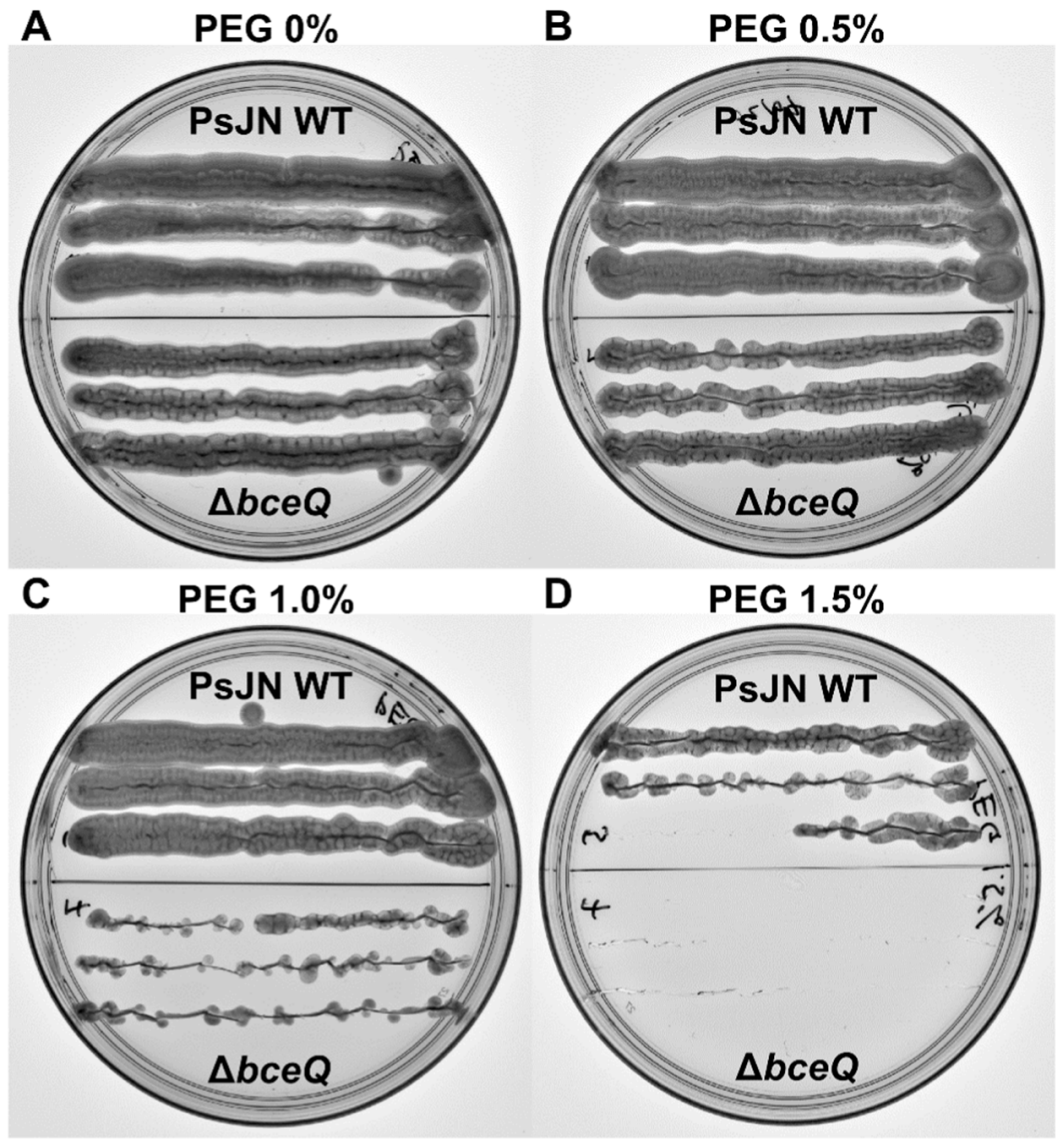
Survival of *Paraburkholderia phytofirmans* PsJN and its ∆*bceQ* mutant under osmotic stress induced by polyethylene glycol (PEG). **(A)** Growth of wild type PsJN and ∆*bceQ* mutant on potato dextrose agar (PDA) without PEG. **(B–D)** Growth of wild type PsJN and ∆*bceQ* mutant on PDA supplemented with 0.5% PEG **(B)**, 1% PEG **(C)**, and 1.5% PEG **(D)**. Each plate was divided into two sections, the top section contained colonies of the wild type PsJN and the bottom part contained colonies of ∆*bceQ* mutant. Photographs were taken at 3 days after inoculation. The experiment was repeated three times with similar results.

Previously it has been shown that EPS plays a role in PsJN tolerance to desiccation (Fu and Yan, 2023). To further explore the role of EPS in PsJN-mediated drought tolerance of pea plant, we investigated whether EPS plays a role in tolerance of PsJN to osmotic stress caused by PEG. PsJN and ∆*bceQ* mutant had a very different survival rate when exposed to PEG treatments. PsJN grew well on PDA supplemented with PEG up to the concentration of 1% (v/v), while at PEG 1.5% (v/v) the growth is not as vigorous (Figure 5). In contrast, ∆*bceQ* mutant grew very poorly on PDA with 1% (v/v) PEG, and no growth was observed at PEG 1.5% (v/v) (Figure 5D). Even at PEG 0.5% ∆*bceQ* mutant growth was not as robust as PsJN (Figure 5B).

### 3.6 Expression of EPS genes were induced by glucose

**Figure 6:**
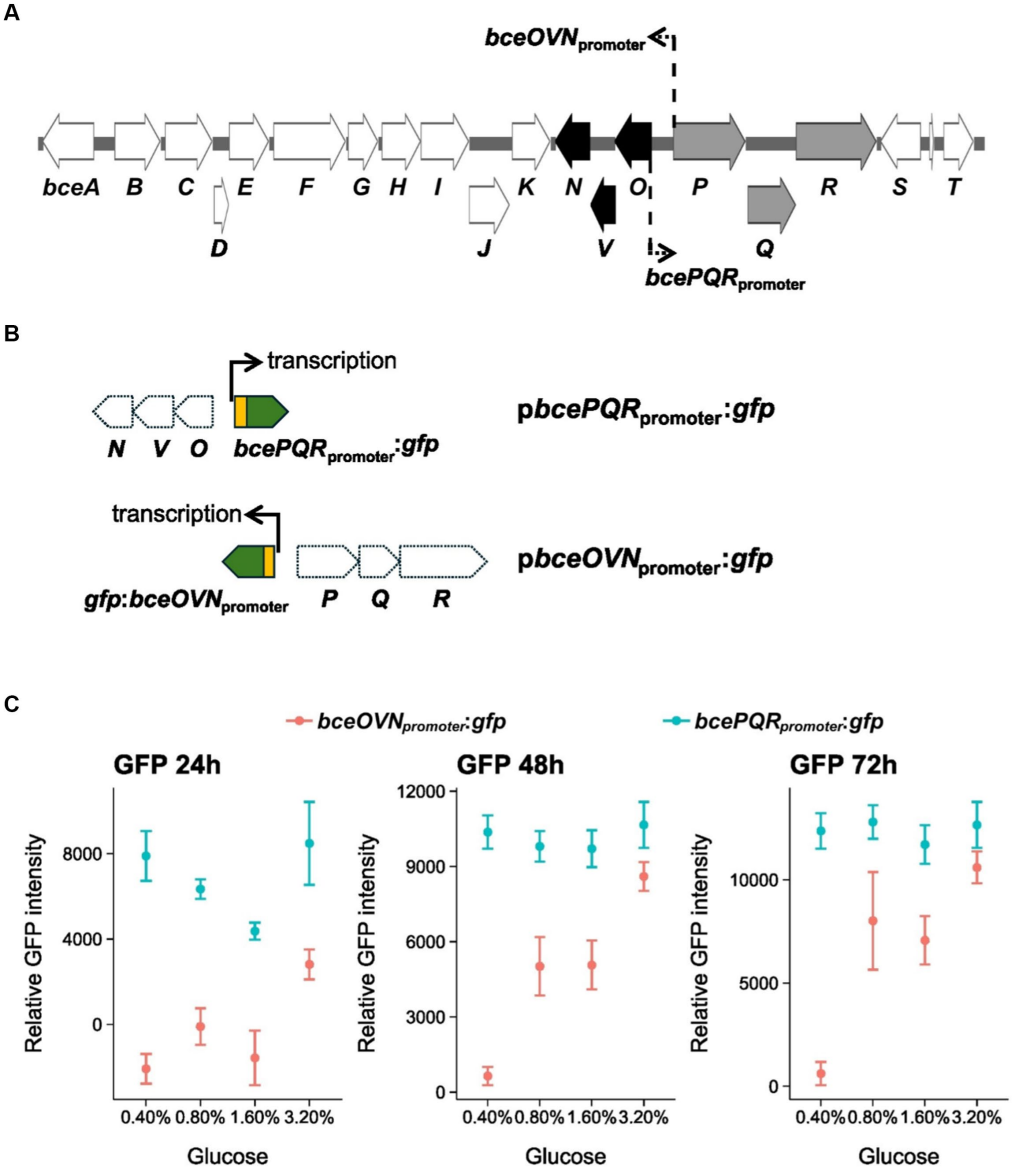
Expression of *bceOVN* and *bcePQR* operons of the *bce* gene cluster under osmotic stress conditions. **(A)** The EPS biosynthesis gene cluster of PsJN. The *bcePQR* operon and the *bceOVN* operon are shown in gray and black, respectively. The location and direction of the promoters of these two operons are shown by line and arrows. **(B)** The *promoter*::*gfp* fusions of the transcriptional reporter constructs used in this work are also shown. **(C)** Expression profile of the *bcePQR* promoter and the *bceOVN* promoter of the EPS biosynthesis gene cluster. The bacterial reporter strains were grown on M9 minimal medium plates with different concentrations of glucose. The relative GFP activity of the reporter strains was calculated by recording the GFP intensity and normalized against the cell density (OD_600_) at 24, 48, and 72 h after inoculation on the culture plates. Data represent the mean values of four replicates. The experiments were repeated two times independently. Thin, dashed arrows in panel **(A)** indicate locations and directions of the *bcePQR* and *bceOVN* promoters.

Gene *bceQ* locates in the *bcePQR* transcriptional operon which is a part of the EPS biosynthesis gene cluster of PsJN (Figure 6A). Root exudates have diverse sugars and carbohydrates that may influence gene expression for EPS production. To understand the expression profiles of *bceQ*, the promoter of the *bcePQR* transcriptional operon was fused with a promoterless *gfp*. The generated reporter construct, p*bcePQR*_promoter_:*gfp* (Figure 6B), was transferred into the wild type PsJN. The reporter strain was cultured on M9 medium plates with different concentrations of glucose, one of the major sugars in root and seed exudates. Then GFP activity of the reporter strain was measured at different time points.

Results show that overall GFP activity of the *bcePQR*_promoter_:*gfp* transcriptional reporter fusion did not change remarkably when the reporter strain was cultured with different concentrations of glucose (Figure 6C), indicating that expression of the *bcePQR*_promoter_ is not regulated by glucose.

The *bce* gene cluster contains another operon, *bceOVN*, that is adjacent to the *bcePQR* operon with an opposite transcriptional orientation (Figure 6A). To understand if expression of the *bceOVN* operon is regulated by glucose, a *bceOVN*_promoter_:*gfp* transcriptional reporter fusion was made and transferred into wild type PsJN (Figure 6B). As shown in Figure 6C, expression of the *bceOVN*_promoter_:*gfp* reporter fusion was significantly induced by adding glucose to the culture medium. We observed around 10-fold activation of *bceOVN*_promoter_:*gfp* expression at glucose 0.8 and 1.6% at 48 and 72 h of incubation compared with at glucose 0.4%. At glucose 3.2%, the induction was almost 20-fold than at glucose 0.4%. Overall, these results show that expression of the *bceOVN* operon, but not the *bcePQR* transcriptional operon, of the EPS biosynthesis gene cluster can be induced by the presence of glucose.

## 4 Discussion

The present study demonstrates that wild type PsJN promotes pea seed germination and seedling growth under drought conditions. We also show that EPS is required for wild type PsJN to confer the beneficial effects to pea plants. EPS plays a role in the survival of wild type PsJN to osmotic stress and expression study shows that expression of the EPS biosynthesis genes is induced by glucose, which is one of the major sugars in seed and root exudates. These data suggest a possible mechanism of how EPS contributes to the PsJN-mediated beneficial effects.

Our results show that EPS is required for PsJN to effectively colonize pea roots under drought conditions. Although some hints of the mechanisms by which EPS play a role in root surface attachment and colonization of beneficial bacteria have been proposed (Meneses et al., 2011; Balsanelli et al., 2014; Liu et al., 2020), and PsJN has been shown to induce drought tolerance (Naveed et al., 2014a,b; Sheibani-Tezerji et al., 2015), how EPS contributes to root colonization and drought tolerance by PsJN is previously unknown. The correlation between reduced colonization ability and drought tolerance due to the loss of EPS has been shown in *B. amyloliquefaciens* FZB42 (Lu et al., 2018). However, our data show that EPS is required for not only plant colonization, the survival of PsJN under drought stress also requires EPS. This suggests that EPS is required for both plant colonization and bacterial cell survival under drought stress. The impaired colonization and survival of wild type PsJN due to the loss of EPS are probably the main reasons why the ∆*bceQ* mutant could not express the beneficial functions like the wild-type. The role of EPS in root colonization may not be universal as in the case with *Herbaspirillum seropedicae*, in which although the EPS mutant are unable to form biofilm, they are still able to colonize plant (Balsanelli et al., 2014). This suggests that other factors may compensate for the lack of EPS to achieve plant colonization. However, the requirement of EPS for PsJN to confer beneficial effects to plant under drought stress conditions can be explained, at least partially, by the importance of EPS in the colonization of PsJN on plant surfaces and the bacterial tolerance to stressful conditions.

PsJN colonization on roots progresses in distinguishable stages by which colonization shifts from proximal region (mature zone) of the roots to the distal region (elongation/tip zone) over time. This finding suggests that PsJN colonizes roots with the distribution oriented in the same direction as the growing direction of the roots. The relative abundance of PsJN at the root tips has also been reported in *Vitis vinifera* (Compant et al., 2005). Sites with high exudation are possible colonization hotspots that would attract bacteria and it is generally agreed that the exudation rates are high in the elongation zone rather than in the mature root zones. Presumably, PsJN actively migrated following the changed root exudation sites. Drought stress can significantly affect the composition of root exudate metabolome (Gargallo-Garriga et al., 2018; Lin et al., 2023). These changes in turn can select for specific bacteria that enhance drought protection. Glucose is one of the major sugars in root exudates and accumulates in response to drought stresses (Gargallo-Garriga et al., 2018). Interestingly, we show that glucose induces the expression of the EPS biosynthesis genes, in this case the *bceOVN* operon, in wild type PsJN. The *bceO*, *bceV*, and *bceN* genes in the *bceOVN* operon encode acyltransferases and mannose dehydratase, respectively, which are enzymes involved in the biosynthesis of EPS. On the other hand, the *bceP*, *bceQ*, and *bceR* encode beta propeller protein domain, transmembrane domain of flippase, and Rossmann-like domain of glycosyltransferase, respectively, which are mostly structural proteins involved in transport of EPS. The *bcePQR* operon was not induced by glucose but constitutively expressed at different concentrations of glucose (Figure 6). This result suggests genes of the *bcePQR* operon are important for EPS production, which is consistent with the observations that mutation of the *becQ* gene decreased the bacterial survival under stress condition (Figure 5) and compromised the beneficial effects on pea plant (Figure 4). In another study, organic acids were found to accumulate in maize during drought stress and serve as effective chemo-attractants to the beneficial bacterium *B. subtilis* (Williams and de Vries, 2020). Furthermore, exudate metabolic changes caused by abiotic stresses can also alter the EPS composition of bacteria. For example, EPS composition of *Azospirillum brasilense* was changed by wheat root exudates under saline stress and this altered EPS profile is presumably necessary for the bacterium to adapt to the stress and thus aids colonization process under the stress condition (Fischer et al., 2003). We presume that the dynamics in PsJN colonization on pea roots under drought stress can be attributed to any of the above possibilities and this outstanding question needs to be addressed to advance our understanding of how PsJN colonizes root under drought stress and the consequences of this colonization pattern on its capacity to promote drought tolerance.

Interestingly, not only that wild type PsJN was able to effectively colonize and stimulate growth of pea under drought conditions, but it also stimulated the formation of nodules. This leads to conjecture that effective colonization of roots by wild type PsJN under drought stress maintains the nodulation status of pea. There are three possible mechanisms to explain this. First, direct interaction between PsJN and rhizobia facilitates symbiosis between pea and rhizobia and thus the formation of nodules. Second possibility is through indirect interaction between PsJN and rhizobia, in which the symbiosis between PsJN and pea alleviates drought stress and promotes growth of pea and this leads to improved overall growth and health of pea, which in turn favors nodule symbiosis. Thirdly, symbiosis between PsJN and pea generates PsJN-derived signaling in plants that attracts rhizobia to form symbiosis with pea. In the *Rhizobium*-legume symbiosis, bacterial EPS are essential for the formation of the infection thread, for nodule development (Kannenberg and Brewin, 1994). Apart from rhizobia, legume nodules harbor nodule-associated bacteria (NAB), some of which includes bacteria from the genus of *Paraburkholderia*, that co-existence of these NAB with rhizobia in roots can significantly increases nodulation and overall growth (Moulin et al., 2014; Pang et al., 2021; Youseif et al., 2021). It is not known whether PsJN possesses the traits as a NAB although the genome sequence of PsJN does not carry genes for nitrogen fixation (*nif* genes) and it has not been shown to be able to fix nitrogen (Mitter et al., 2013; Lowman et al., 2016). Future effort is needed to determine the mechanism of how PsJN stimulates nodule formation under drought stress.

## Data availability statement

The raw data supporting the conclusions of this article will be made available by the authors, without undue reservation.

## Author contributions

CP: Conceptualization, Data curation, Formal analysis, Investigation, Methodology, Software, Validation, Writing – original draft, Writing – review & editing. QY: Conceptualization, Data curation, Formal analysis, Funding acquisition, Investigation, Methodology, Project administration, Resources, Software, Supervision, Validation, Writing – review & editing.

## References

[ref1] Ait Barka E. Nowak J. Clément C. (2006). Enhancement of chilling resistance of inoculated grapevine plantlets with a plant growth-promoting rhizobacterium, *Burkholderia phytofirmans* strain PsJN. Appl. Environ. Microbiol. 72:11. doi: 10.1128/AEM.01047-06 PMC1636148 16980419

[ref2] Balsanelli E. de Baura V. A. Pedrosa Fde O. de Souza E. M. Monteiro R. A. (2014). Exopolysaccharide biosynthesis enables mature biofilm formation on abiotic surfaces by *Herbaspirillum seropedicae*. PLoS One. 9, 1–10. doi: 10.1371/journal.pone.0110392 PMC4195743 25310013

[ref3] Chandra D. Srivastava R. Glick B. R. Sharma A. K. (2018). Drought-tolerant *Pseudomonas* spp. improve the growth performance of finger millet (*Eleusine coracana* (L.) Gaertn.) under non-stressed and drought-stressed conditions. Pedosphere 28:2. doi: 10.1016/S1002-0160(18)60013-X

[ref4] Compant S. Reiter B. Sessitsch A. Nowak J. Clément C. Barka E. A. (2005). Endophytic colonization of *Vitis vinifera* L. by plant growth-promoting bacterium *Burkholderia* sp. strain PsJN. Appl. Environ. Microbiol. 71:4. doi: 10.1128/AEM.71.4.1685-1693.2005 PMC1082517 15811990

[ref5] Donoso R. Leiva-Novoa P. Zúñiga A. Timmermann T. Recabarren-Gajardo G. González B. (2016). Biochemical and genetic bases of indole-3-acetic acid (auxin phytohormone) degradation by the plant-growth-promoting rhizobacterium *Paraburkholderia phytofirmans* PsJN. Appl. Environ. Microbiol. 83:e01991. doi: 10.1128/AEM.01991-16, PMID: 27795307 PMC5165117

[ref6] Edwards J. Johnson C. Santos-Medellín C. Lurie E. Podishetty N. K. Bhatnagar S.. (2015). Structure, variation, and assembly of the root-associated microbiomes of rice. Proc. Natl. Acad. Sci. 112, E911–E920. doi: 10.1073/pnas.1414592112, PMID: 25605935 PMC4345613

[ref7] Esmaeel Q. Miotto L. Rondeau M. Leclère V. Clément C. Jacquard C.. (2018). *Paraburkholderia phytofirmans* PsJN-plants interaction: From perception to the induced mechanisms. Front. Microbiol. 9:2093. doi: 10.3389/fmicb.2018.02093, PMID: 30214441 PMC6125355

[ref8] Fischer S. E. Miguel M. J. Mori G. B. (2003). Effect of root exudates on the exopolysaccharide composition and the lipopolysaccharide profile of *Azospirillum brasilense* Cd under saline stress. FEMS Microbiol. Lett. 219, 53–62. doi: 10.1016/S0378-1097(02)01194-1, PMID: 12594023

[ref9] Fu B. Yan Q. (2023). Exopolysaccharide is required for motility, stress tolerance, and plant colonization by the endophytic bacterium *Paraburkholderia phytofirmans* PsJN. Front. Microbiol. 14:1218653. doi: 10.3389/fmicb.2023.1218653, PMID: 37670984 PMC10475733

[ref10] Gargallo-Garriga A. Preece C. Sardans J. Oravec M. Urban O. Peñuelas J. (2018). Root exudate metabolomes change under drought and show limited capacity for recovery. Sci. Rep. 8:12696. doi: 10.1038/s41598-018-30150-0, PMID: 30140025 PMC6107494

[ref11] Issa A. Esmaeel Q. Sanchez L. Courteaux B. Guise J.-F. Gibon Y.. (2018). Impacts of *Paraburkholderia phytofirmans* strain PsJN on tomato (*Lycopersicon esculentum* L.) under high temperature. Front. Plant Sci. 9:1397. doi: 10.3389/fpls.2018.01397, PMID: 30405648 PMC6201190

[ref12] Janczarek M. Rachwał K. Cieśla J. Ginalska G. Bieganowski A. (2015). Production of exopolysaccharide by *Rhizobium leguminosarum* bv. *trifolii* and its role in bacterial attachment and surface properties. Plant Soil 388, 211–227. doi: 10.1007/s11104-014-2320-5

[ref13] Kannenberg E. L. Brewin N. J. (1994). Host-plant invasion by Rbizobium: The role of cell-surface components. Trends Microbiol. 2, 277–283. doi: 10.1016/0966-842X(94)90004-3, PMID: 7981970

[ref14] Kavamura V. N. Santos S. N., Silva, J. L. da, Parma M. M. Ávila L. A. Visconti A. Zucchi T. D. Taketani R. G. Andreote F. D., & Melo, I. S. de. (2013). Screening of Brazilian cacti rhizobacteria for plant growth promotion under drought. Microbiol. Res. 168, 183–191. doi: 10.1016/j.micres.2012.12.002, PMID: 23279812

[ref15] King E. O. Ward M. K. Raney D. E. (1954). Two simple media for the demonstration of pyocyanin and fluorescin. J. Lab. Clin. Med. 44:2. 13184240

[ref16] Kumar M. Mishra S. Dixit V. Kumar M. Agarwal L. Chauhan P. S.. (2016). Synergistic effect of *Pseudomonas putida* and *Bacillus amyloliquefaciens* ameliorates drought stress in chickpea (*Cicer arietinum* L.). Plant Signal. Behav. 11:e1071004. doi: 10.1080/15592324.2015.1071004, PMID: 26362119 PMC4871671

[ref17] Lin H.-A. Coker H. R. Howe J. A. Tfaily M. M. Nagy E. M. Antony-Babu S.. (2023). Progressive drought alters the root exudate metabolome and differentially activates metabolic pathways in cotton (*Gossypium hirsutum*). Front. Plant Sci. 14:1244591. doi: 10.3389/fpls.2023.1244591, PMID: 37711297 PMC10499043

[ref18] Lindow S. Koutsoukis R. Meyer K. Baccari C. (2024). Control of Pierce’s disease of grape with *Paraburkholderia phytofirmans* PsJN in the field. Phytopathology 114, 503–511. doi: 10.1094/PHYTO-06-23-0219-R, PMID: 37913631

[ref19] Liu Y. Bellich B. Hug S. Eberl L. Cescutti P. Pessi G. (2020). The exopolysaccharide cepacian plays a role in the establishment of the *Paraburkholderia phymatum*–*Phaseolus vulgaris* symbiosis. Front. Microbiol. 11:1600. doi: 10.3389/fmicb.2020.01600, PMID: 32765457 PMC7378592

[ref20] Lowman S. Kim-Dura S. Mei C. Nowak J. (2016). Strategies for enhancement of switchgrass (*Panicum virgatum* L.) performance under limited nitrogen supply based on utilization of N-fixing bacterial endophytes. Plant Soil 405, 47–63. doi: 10.1007/s11104-015-2640-0

[ref21] Lu X. Liu S.-F. Yue L. Zhao X. Zhang Y.-B. Xie Z.-K.. (2018). *Epsc* involved in the encoding of exopolysaccharides produced by *Bacillus amyloliquefaciens* FZB42 act to boost the drought tolerance of *Arabidopsis thaliana*. Int. J. Mol. Sci. 19, 1–18. doi: 10.3390/ijms19123795, PMID: 30501023 PMC6320885

[ref22] Mahmoudi T. R. Yu J. M. Liu S. Pierson L. S. III Pierson E. A. (2019). Drought-stress tolerance in wheat seedlings conferred by phenazine-producing rhizobacteria. Front. Microbiol. 10:1590. doi: 10.3389/fmicb.2019.01590, PMID: 31354678 PMC6636665

[ref23] Meneses C. H. Rouws L. F. Simões-Araújo J. L. Vidal M. S. Baldani J. I. (2011). Exopolysaccharide production is required for biofilm formation and plant colonization by the nitrogen-fixing endophyte *Gluconacetobacter diazotrophicus*. Mol. Plant-Microbe Interact. 24, 1448–1458. doi: 10.1094/MPMI-05-11-0127 21809982

[ref8001] Miller W. G. Leveau J. H. Lindow S. E. (2000). Improved *gfp* and *inaZ* broad-host-range promoter-probe vectors. Mol. Plant-Microbe Interact. 13, 1243–1250. doi: 10.1094/MPMI.2000.13.11.1243 11059491

[ref24] Mitter B. Petric A. Shin M. W. Chain P. S. Hauberg-Lotte L. Reinhold-Hurek B.. (2013). Comparative genome analysis of *Burkholderia phytofirmans* PsJN reveals a wide spectrum of endophytic lifestyles based on interaction strategies with host plants. Front. Plant Sci. 4:120. doi: 10.3389/fpls.2013.00120 23641251 PMC3639386

[ref25] Moulin L. Klonowska A. Caroline B. Booth K. Vriezen J. A. C. Melkonian R.. (2014). Complete genome sequence of *Burkholderia phymatum* STM815(T), a broad host range and efficient nitrogen-fixing symbiont of Mimosa species. Stand. Genomic Sci. 9, 763–774. doi: 10.4056/sigs.4861021, PMID: 25197461 PMC4148976

[ref26] Naveed M. Hussain M. B. Zahir Z. A. Mitter B. Sessitsch A. (2014a). Drought stress amelioration in wheat through inoculation with *Burkholderia phytofirmans* strain PsJN. Plant Growth Regul. 73, 121–131. doi: 10.1007/s10725-013-9874-8

[ref27] Naveed M. Mitter B. Reichenauer T. G. Wieczorek K. Sessitsch A. (2014b). Increased drought stress resilience of maize through endophytic colonization by *Burkholderia phytofirmans* PsJN and *Enterobacter* sp. FD17. Environ. Exp. Bot. 97, 30–39. doi: 10.1016/j.envexpbot.2013.09.014

[ref28] Pang J. Palmer M. Sun H. J. Seymour C. O. Zhang L. Hedlund B. P.. (2021). Diversity of root nodule-associated bacteria of diverse legumes along an elevation gradient in the Kunlun Mountains, China. Front. Microbiol. 12:633141. doi: 10.3389/fmicb.2021.633141, PMID: 33664721 PMC7920992

[ref29] Pieterse C. M. J. Van der Does D. Zamioudis C. Leon-Reyes A. Van Wees S. C. M. (2012). Hormonal modulation of plant immunity. Annu. Rev. Cell Dev. Biol. 28, 489–521. doi: 10.1146/annurev-cellbio-092910-154055 22559264

[ref30] Pieterse C. M. J. Zamioudis C. Berendsen R. L. Weller D. M. Van Wees S. C. Bakker P. A. (2014). Induced systemic resistance by beneficial microbes. Annu. Rev. Phytopathol. 52, 347–375. doi: 10.1146/annurev-phyto-082712-102340 24906124

[ref31] Rodríguez-Navarro D. N. Dardanelli M. S. Ruíz-Saínz J. E. (2007). Attachment of bacteria to the roots of higher plants. FEMS Microbiol. Lett. 272, 127–136. doi: 10.1111/j.1574-6968.2007.00761.x 17521360

[ref32] Sarma R. K. Saikia R. (2014). Alleviation of drought stress in mung bean by strain *Pseudomonas aeruginosa* GGRJ21. Plant Soil 377:111. doi: 10.1007/s11104-013-1981-9

[ref33] Schmid J. Sieber V. Rehm B. (2015). Bacterial exopolysaccharides: Biosynthesis pathways and engineering strategies. Front. Microbiol. 6:496. doi: 10.3389/fmicb.2015.00496, PMID: 26074894 PMC4443731

[ref34] Sheibani-Tezerji R. Rattei T. Sessitsch A. Trognitz F. Mitter B. (2015). Transcriptome profiling of the endophyte *Burkholderia phytofirmans* PsJN indicates sensing of the plant environment and drought stress. MBio 6:e00621. doi: 10.1128/mBio.00621-15, PMID: 26350963 PMC4600099

[ref35] Su F. Jacquard C. Villaume S. Michel J. Rabenoelina F. Clément C.. (2015). *Burkholderia phytofirmans* PsJN reduces impact of freezing temperatures on photosynthesis in *Arabidopsis thaliana*. Front. Plant Sci. 6:810. doi: 10.3389/fpls.2015.00810 26483823 PMC4591482

[ref36] Sun Y. Cheng Z. Glick B. R. (2009). The presence of a 1-aminocyclopropane-1-carboxylate (ACC) deaminase deletion mutation alters the physiology of the endophytic plant growth-promoting bacterium *Burkholderia phytofirmans* PsJN. FEMS Microbiol. Lett. 296, 131–136. doi: 10.1111/j.1574-6968.2009.01625.x, PMID: 19459964

[ref37] Timmermann T. Armijo G. Donoso R. Seguel A. Holuigue L. González B. (2017). *Paraburkholderia phytofirmans* PsJN protects *Arabidopsis thaliana* against a virulent strain of *Pseudomonas syringae* through the activation of induced resistance. Mol. Plant-Microbe Interact. 30, 215–230. doi: 10.1094/MPMI-09-16-0192-R 28118091

[ref38] Trong N.-H. Doré J. Gaucher M. Jacquard C. Richet N. Leclère V.. (2022). Biological control of grapevine crown gall disease, caused by *Allorhizobium vitis*, using *Paraburkholderia phytofirmans* PsJN. PhytoFrontiers 2, 391–403. doi: 10.1094/PHYTOFR-03-22-0018-R

[ref39] Weilharter A. Mitter B. Shin M. V. Chain P. S. G. Nowak J. Sessitsch A. (2011). Complete genome sequence of the plant growth-promoting endophyte *Burkholderia phytofirmans* strain PsJN. J. Bacteriol. 193, 3383–3384. doi: 10.1128/jb.05055-11, PMID: 21551308 PMC3133278

[ref40] Williams A. de Vries F. T. (2020). Plant root exudation under drought: Implications for ecosystem functioning. New Phytol. 225, 1899–1905. doi: 10.1111/nph.16223, PMID: 31571220

[ref41] Yang A. Akhtar S. S. Fu Q. Naveed M. Iqbal S. Roitsch T.. (2020). *Burkholderia phytofirmans* PsJN stimulate growth and yield of quinoa under salinity stress. Plan. Theory 9, 1–16. doi: 10.3390/plants9060672 PMC7355930 32466435

[ref42] Youseif S. H. Abd El-Megeed F. H. Abdelaal A. S. Ageez A. Martínez-Romero E. (2021). Plant–microbe–microbe interactions influence the faba bean nodule colonization by diverse endophytic bacteria. FEMS Microbiol. Ecol. 97:fiab138. doi: 10.1093/femsec/fiab138, PMID: 34610117

[ref43] Zúñiga A. Poupin M. J. Donoso R. Ledger T. Guiliani N. Gutiérrez R. A.. (2013). Quorum sensing and indole-3-acetic acid degradation play a role in colonization and plant growth promotion of *Arabidopsis thaliana* by *Burkholderia phytofirmans* PsJN. Mol. Plant-Microbe Interact. 26, 546–553. doi: 10.1094/MPMI-10-12-0241-R 23301615

